# Glutamine addiction in tumor cell: oncogene regulation and clinical treatment

**DOI:** 10.1186/s12964-023-01449-x

**Published:** 2024-01-03

**Authors:** Xian Li, Xueqiang Peng, Yan Li, Shibo Wei, Guangpeng He, Jiaxing Liu, Xinyu Li, Shuo Yang, Dai Li, Weikai Lin, Jianjun Fang, Liang Yang, Hangyu Li

**Affiliations:** Department of General Surgery, The Fourth Affiliated Hospital, China Medical University, Shenyang, 110032 China

**Keywords:** Glutamine, Metabolism, Tumor, Oncogene clinical treatment

## Abstract

After undergoing metabolic reprogramming, tumor cells consume additional glutamine to produce amino acids, nucleotides, fatty acids, and other substances to facilitate their unlimited proliferation. As such, the metabolism of glutamine is intricately linked to the survival and progression of cancer cells. Consequently, targeting the glutamine metabolism presents a promising strategy to inhibit growth of tumor cell and cancer development. This review describes glutamine uptake, metabolism, and transport in tumor cells and its pivotal role in biosynthesis of amino acids, fatty acids, nucleotides, and more. Furthermore, we have also summarized the impact of oncogenes like *C-MYC*, *KRAS*, *HIF*, and *p53* on the regulation of glutamine metabolism and the mechanisms through which glutamine triggers mTORC1 activation. In addition, role of different anti-cancer agents in targeting glutamine metabolism has been described and their prospective applications are assessed.

## Introduction

**Fig. 1:**
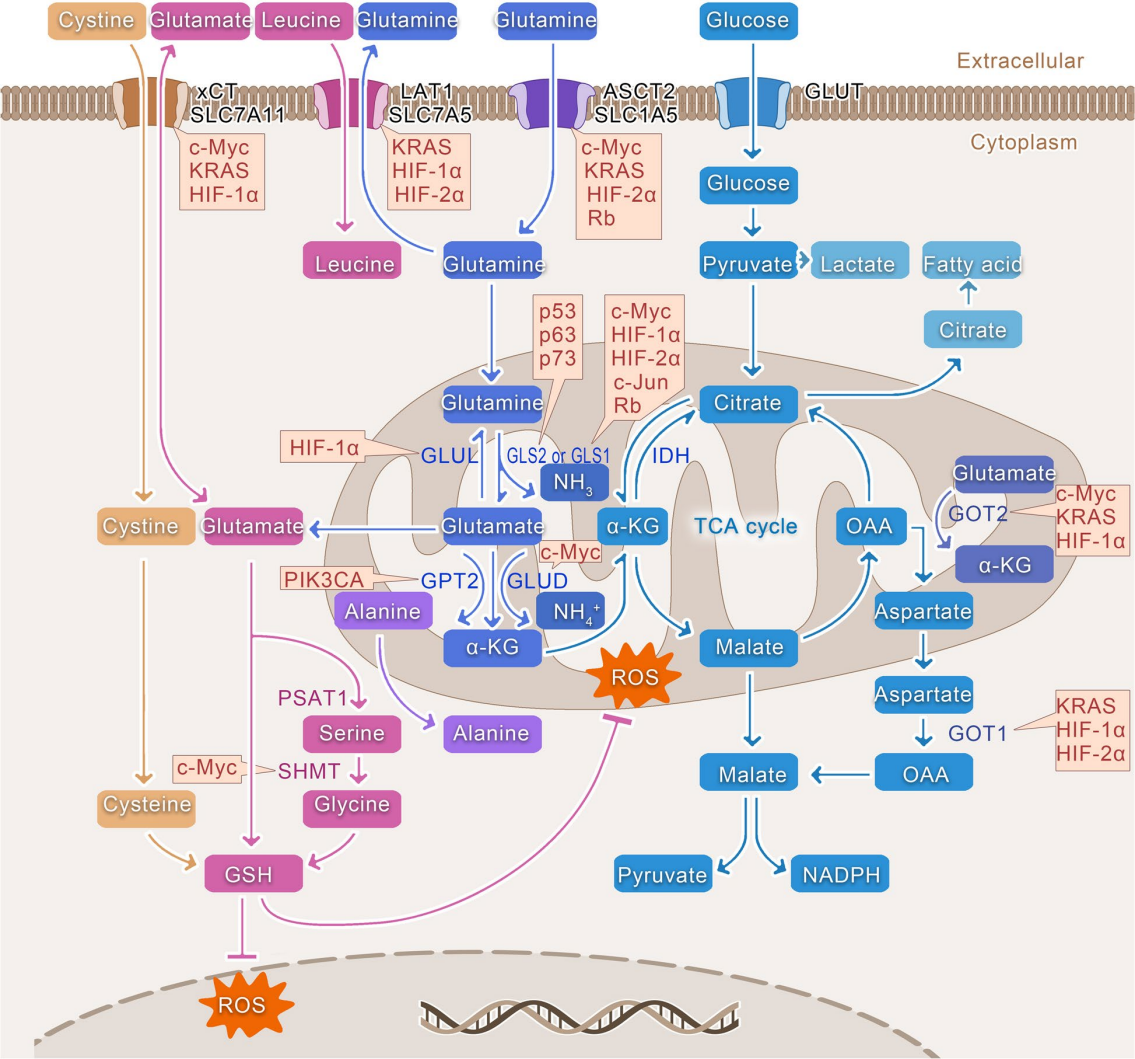
Glutamine metabolism, synthesis, and regulation by oncogenes. *SLC1A5* (*ASCT2*) facilitates the passage of glutamine across the membrane. Thereafter, glutamine is converted to glutamate by GLS, and NH_3_ is released. Glutamate is then converted to α-KG by *GLUD1* or other mitochondrial aminotransferases, such as *GPT2* and *GOT2*. α-KG can then enter the TCA cycle through anaplerosis, thereby providing various intermediates for amino acid and lipid synthesis. Glutamine-related cysteine synthesizes glutathione with glutamate, cysteine, and glycine. Subsequently, glutathione can scavenge ROS both within the mitochondria and the nucleus. Various oncogenes have been identified to regulate different sites in glutamine metabolic processes, as indicated by the yellow boxes

Although tumors may differ in their origin and characteristics, they undergo metabolic reprogramming due to genetic mutations and altered cell signalling. This reprogramming alters their energy metabolism, allowing them to better adapt to nutrient-deprived environments, thereby meeting the demand for energy and material resources for achieving continuous, rapid and uncontrolled proliferation [1]. One of the most representative alterations to metabolic processes in tumor cells, which was first described by Otto Warburg, involves increased glucose uptake and consumption, even under aerobic conditions, thereby driving cancer cells to convert glucose preferentially to pyruvate and then to lactate, a process known as aerobic glycolysis, or the Warburg effect [2–5]. This process leads to a reduction in the amount of pyruvate that enters the tricarboxylic acid (TCA) cycle (also known as the Krebs cycle). One notable factor is that the fast-growing cancer cells heavily rely on the “naplerotic” mechanism to introduce α-ketoglutarate derived from glutamine into the TCA cycle. This compensates for the deficiencies in TCA cycle intermediates caused by aerobic glycolysis. Additionally, after decomposition, glutamine is utilized for the synthesis of biological macromolecules. Thus, tumor cells can acquire an adequate supply of biosynthetic intermediates for synthesizing proteins, fatty acids, and nucleotides, through these two routes [6–8] (Fig. 1). Glutamine deprivation in certain tumor cells can lead to growth arrest or even death [9–13], this phenomenon of tumor cells is one of known material addiction [7, 14]. The close relationship between glutamine and tumor cell growth, metastasis and invasion has been a subject of research for several decades, with significant focus on exploring the potential of targeting glutamine metabolic pathways for effective tumor treatment.

In this compressive review, we describe in detail the intracellular metabolic process and biosynthetic role of glutamine and discuss the impact of well-known oncogenes on the regulation of glutamine metabolism. In addition, we have summarized the current status of research related to drugs targeting glutamine metabolic processes.

## Function and metabolism of glutamine

**Fig. 2:**
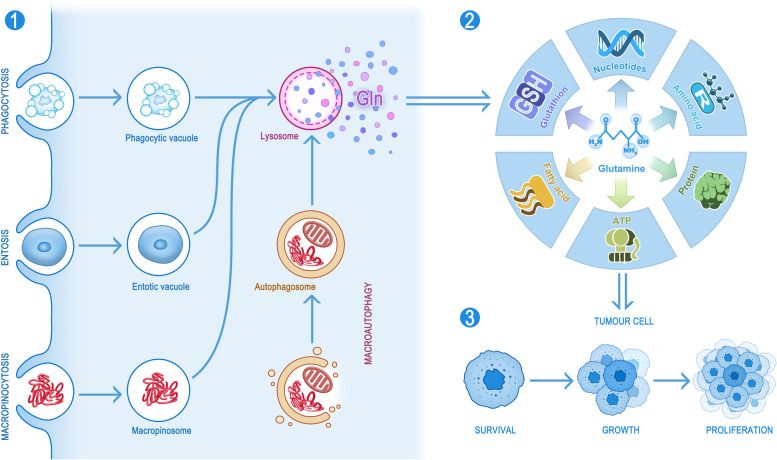
Sources, biosynthesis, and effects of glutamine. Tumor cells have the ability to take up glutamine from extracellular fluids and synthesize de novo glutamine intracellularly (not shown in the figure). Tumor cells can also use two distinct layers of membrane structures to encapsulate intracellular proteins and organelles, which are then fused with the lysosomes and digested to release glutamine. In addition, some tumor cells can also deliver proteins or cells to the lysosomes for digestion and subsequently release glutamine through the processes of macropinocytosis, entosis and phagocytosis (1). The glutamine taken up by the tumor cells can be effectively used to synthesize fatty acids, ATP, proteins, amino acids, nucleotides and glutathione (2), which will ultimately be used for the survival, growth and reproduction of the tumor cells (3)

Glutamine, the most abundant amino acid in both human plasma and muscle, accounts for approximately 20% of the free amino acid pool in blood and 40% of free amino acids in muscle [15]. Glutamine consists of two amino groups: an α-amino group and a terminal amide group that can be easily hydrolysed, resulting in the formation of glutamate and ammonia. These properties enable glutamine to function as both a carbon and nitrogen donor. Glutamine synthesis occurs primarily in the skeletal muscle [16], the brain as well as fat tissues, whereas catabolism occurs mainly in the intestine, liver and kidneys [17, 18]. Glutamine can be synthesized de novo by the body and hence classified as a non-essential amino acid (NEAA). However, in humans with cancer, there is a significant increase in the demand for glutamine by the tumor cells. As a result, maintaining high levels of glutamine becomes crucial, making it a conditionally essential amino acid [19–21]. Specifically, within ovarian cancer cells, glutamine serves as the primary energy source, surpassing the metabolism rates of other amino acids, while glucose does not play a significant role [22–24]. The required amount of necessary glutamine is at least 10-fold higher than that of any other amino acid [6, 25–27]. Cancer cells consume even more glutamine than is needed to meet their energy requirements [28]. Within tumor cells, the de novo synthesis of glutamine can be facilitated by glutamate using glutamine synthetase (GS). Under conditions of limited glutamine, cells can phagocytose various proteins and dysfunctional organelles through a process called macroautophagy and replenish glutamine after digestion by the lysosomes. Extracellular glutamine can be transported into a cell through the plasma membrane transport proteins such as SLC1A5, SLC6A14, and SLC7A6 [29, 30]. Furthermore, under conditions of nutrient deficiency, tumor cells possess the ability to internalize and degrade extracellular proteins in lysosomes after phagocytosis or macropinocytosis, and entosis. The resulting glutamine released from lysosomes is subsequently used by the cells [31] (Fig. 2). Glutamine can then enter a cell and undergoes a series of metabolic processes (Fig. 1). The key enzyme in glutamine metabolism is glutaminase (GLS), which can effectively convert glutamine to glutamate. Two distinct isoforms of GLS have been reported so far: The GLS in kidney (*GLS1*) is commonly expressed in various normal tissues, whereas the GLS in liver (*GLS2*) is found exclusively in the liver, brain and pancreas [32, 33]. Interestingly, upregulation of GLS1 has been associated with increased tumorigenesis, while *GLS2* expression has been linked more closely with quiescent or differentiated cell states. In other words, *GLS1* has been established as an oncogene, but *GLS2* functions as a tumor suppressor gene [34]. Moreover, in a variety of cancers, including colorectal, prostate, and liver cancers, *GLS1* has been reported to be abnormally highly expressed in tumor cells in comparison with the normal adjacent tissues [35–37], and some studies have even suggested that *GLS1* may serve as a novel biomarker for the pathological diagnosis and prognosis of hepatocellular carcinoma [35]. In addition, extensive studies have demonstrated that inhibition of *GLS1* activity can effectively inhibit the tumor growth and impede its advancement. Consequently, there have been significant advancements in the development of various anti-cancer treatments specifically designed to target *GLS1* [38–40].

## Glutamine biosynthesis in tumor cells

As mentioned above, glutamine plays a pivotal role in tumor cells, and only a clear understanding of its involvement in biosynthesis can lead to a better understanding of the regulatory impact of dysregulated oncogenes in tumor cells. Therefore, this section addresses the role of glutamine in NEAAs, nucleotides, and fatty acids and as well as its involvement in combating the generation of reactive oxygen species (ROS) (Fig. 2).

### NEAA production

As essential components of peptides and proteins, amino acids have been implicated to play a key role in regulating in many cellular activities as well as signalling pathways and are intricately linked to cell fate. The higher energy and metabolic demands triggered by the limitless proliferation of tumor cells primarily depend on specific NEAAs serving as substrates for nucleotide synthesis and supporting cellular redox homeostasis. After being transported into cells, glutamine is converted into glutamate. After being converted into α-ketoglutarate (α-KG), glutamate can enter the TCA cycle through anaplerosis to assist in the oxidative phosphorylation or reductive carboxylation pathways. This process further provides intermediates for amino acid and lipid synthesis (Fig. 1).

In detail, glutamate exchanged for cystine via SLC7A11 (also known as the xCT), which in turn leads to the production of cysteine. *GOT2* transfers nitrogen from glutamate to oxaloacetic acid to generate aspartate. Moreover, under the action of ASNS, a transfer of an amide group occurs from glutamine to aspartate, resulting in the production of. *GPT2* transfers nitrogen from glutamate to pyruvate, producing alanine and α-KG. In contrast, *PSAT1* facilitates the transfer of nitrogen from glutamate to 3-phosphohydroxypyruvate, resulting in the production of phosphoserine and α-KG, consequently generating serine. Subsequently, through SHMT, serine can be converted to glycine. Proline can also be obtained from glutamate through a series of transformations. Moreover, intracellular glutamine can be exchanged for extracellular leucine through SLC7A5 (also known as the LAT1) (Fig. 1). Thus, glutamine plays an important role in maintaining the stability of the amino acid pool in a cell, thereby providing NEAAs, such as aspartate, alanine, and glycine, in proliferating cells (Fig. 1). In vitro experiments have demonstrated that more than one-half of the NEAAs required for protein synthesis in tumor cells were derived from glutamine [41]. Another vital role of aspartate is its contribution as a nitrogen donor for the synthesis of nucleotides, while also serving as a carbon donor for the synthesis of UTP and ATP. Aspartate-transporting proteins are not remarkably effective in mammalian cells, and as a result, the majority of intracellular aspartate is generated through the breakdown of glutamine [42, 43]. The subsequent section provides additional details on the crucial function of glutamine-derived aspartate in the synthesis of nucleotides. In addition, Moreover, the synthesis of glutathione is intricately linked with glutamine and plays an important intracellular role, as described below. Interestingly, tyrosine is the only NEAA that is not derived from glucose or glutamine origin; it is produced directly from the EAA phenylalanine.

Glutamine not only supplies amino acids for protein synthesis but also assists in facilitating protein synthesis by blocking the endoplasmic reticulum (ER) stress pathway and the integrated stress response (ISR) [10, 11, 44, 45].

### Nucleotide production

The nitrogen in glutamine contributes to the de novo biosynthesis of purines and pyrimidines; hence, the catabolism of glutamine is essential for the for the continuous proliferation and division of tumor cells [9, 46–48]. Glutamine-deficient tumor cells undergo cell cycle arrest, which can be re-established by the addition of exogenous nucleotides; but, it is difficult to restore by the addition TCA intermediates [49].

During pyrimidine synthesis in tumor cells, the nitrogen required for two specific enzymatic steps, namely carbamoyl phosphate synthetase II (CPSII) and cytidine triphosphate synthetase (CTPS), is supplied by the amide group of glutamine. One of the two nitrogens contained in the pyrimidine ring is provided by the amide group of glutamine through the CPSII enzyme, whereas the other is derived from aspartic acid. The α-amino group of glutamine is then added to uridine triphosphate to form cytidine triphosphate. Three carbons in the pyrimidine ring are contributed by aspartate but these carbon atoms originate primarily from glutamine not glucose [50], while the other carbon is derived from CO_2_. In addition, during the purine synthesis in tumor cells, the amide group of glutamine provides nitrogen for three of the enzymatic steps mediated by phosphoribosylpyrophosphate (PRPP) aminotransferase, phosphoribosylformylglycinamidine (FGAM) synthase, and guanosine monophosphate (GMP) synthase, respectively. The purine ring contains two nitrogen molecules which originate from glutamine, while the remaining nitrogen molecules are derived from aspartate and glycine as well as from the α-amino group of glutamines. The carbon in the purine ring is derived from glycine and CO_2_ [51].

In addition, glutamine enhances the production of nucleotides in cancerous cells via the aspartate pathway. Aspartate, an important precursor for nucleotide and protein synthesis, is an essential amino acid involved in regulation of tumor cell proliferation. However, the levels of aspartate in human plasma are low, and the SLC1A3 transporter protein does not effectively transport aspartate. Therefore, the aspartate supply in tumor cells is limited [52]. Thus, aspartate produced from glutamine through the TCA cycle and transamination is essential for tumor cells [8, 43, 47, 53, 54]. However, However, an essential function of the mitochondrial electron transport chain (ETC), which is required for cell proliferation, is promotion of aspartate production [42, 55]. The hypoxic microenvironment damages the ETC of tumor cells, which in turn significantly reduces the synthesis level of aspartic acid, affecting the production rate of nucleotides, and thereby inhibiting rate of cell proliferation. The inefficient process of nucleotide production in tumor cells can be compensated by the aspartate generated by glutamine through reductive carboxylation and thus partially restore the nucleotide production in tumor cells [8, 42, 55–57]. On the contrary, *KRAS*-driven tumor cells have the ability to bypass the late G1 checkpoint of the cell cycle [58] and are arrested in the S phase or G2/M phase due to deprivation of glutamine, which is solely responsible for this phenomenon mine. The aspartate derived from glutamine can effectively rescue the tumor cells from stagnating in the S phase and reduces their sensitivity to apoptotic damage, resulting in reduced tumor growth and decreased ability for tumor cell proliferation [49, 58, 59].

It has been proposed that NADPH can be generated during glutamine catabolism by malic enzymes, ultimately promoting nucleotide biosynthesis [53]. The glutamine-dependent mTOR signalling pathway-activated enzymes carbamoyl-phosphate synthetase 2, aspartate transcarbamylase, and dihydroorotase (CAD) can then catalyse the incorporation of glutamine-derived nitrogen into pyrimidine precursors [60–62]. Furthermore, the synthesis of asparagine from glutamine within tumor cells can impact the production of nucleotides by modulating serine levels [61]. In conclusion, these studies have provided valuable insights into the significant contribution of glutamine in facilitating the production of nucleotides during tumor cell proliferation.

### Fatty acid synthesis

Fatty acid synthesis is a critical metabolic pathway responsible for continued proliferation of tumor cells by providing them with various substances required for energy storage, membrane biosynthesis and signal transduction [63]. First, fatty acids can be used to synthesize structural lipids necessary for the proliferation of tumor cells.

At the same time within tumor cells, fatty acids have the ability to produce crucial signalling lipids that act as indispensable signalling molecules. For instance, cellular processes like inflammation, cell migration, and tumor survival are influenced by bioactive lipids such as sphingosine-1-phosphate and lysophosphatidic acid. Additionally, the abundance and saturation levels of cellular fatty acids can also determine the activity of signalling proteins that require acylation, such as the WNT protein, which is often dysregulated in tumor cells [64]. Interestingly, the synthesis and modification of fatty acids can also determine the composition of the membrane lipids from which they are derived, thereby affecting the function of membrane-containing organelles in tumor cells. For example, cardiolipins are structurally unique phospholipids primarily located in the inner membrane of mitochondria [65]. Moreover, by affecting the synthesis of this specific type of lipid, fatty acids can potentially regulate ETC activity in thus mitochondria, thus directly affecting cellular bioenergetics of tumor cells [66]. Furthermore, enhanced metabolism of fatty acids in cancerous cells can alter the saturation levels of lipids in the cell membranes. This is achieved by increasing the relative content of saturated and monounsaturated substances and reducing the relative content of polyunsaturated substances [67]. This higher saturation level offers protection to cancer cells against ROS damage as saturated membrane lipids are more resistant to peroxidation.

In normal cells, most endogenous fatty acids are synthesized from the citric acid in the TCA cycle. However, tumor cells experience the Warburg effect, which limits the glucose entering the TCA cycle. This leads to a decrease in the production of its intermediate product, citric acid, hindering the conversion of citric acid into fatty acids. Consequently, meeting the fatty acid requirements of tumor cells becomes difficult [68]. Tumor cells under conditions of severe nutrient deficiency and hypoxic microenvironments employ glutamine as an alternative primary carbon source in the TCA cycle to synthesize fatty acids to compensate for the lack of fatty acids [63]. This occurs through the reversal of the TCA cycle by α-KG derived from glutamine under the action of isocitrate dehydrogenase, which leads to the reduction and carboxylation of NADPH to produce citrate. To facilitate fatty acid synthesis, mitochondrial citrate is transported across the inner mitochondrial membrane into the cytosol through the citrate carrier (CIC or SLC25A1). The aforementioned process becomes especially apparent under conditions of hypoxia or mitochondrial defects [69–71] (Fig. 1).

### Fight against ROS

Cells activate complex mechanisms to maintain the balance between ROS and antioxidants, i.e., redox homeostasis, in order to ensure that cells are protected from oxidative stress-derived damage and can maintain normal cellular function. At low or moderate levels, ROS can promote tumorigenesis by acting as signalling molecules or by promoting DNA mutations [72]. However, when ROS levels exceed the redox capacity of the cells, they may cause substantial damage to various macromolecules such as proteins, lipids, and nucleotides, leading to severe cell damage or death [73–75]. However, due to abnormalities in their metabolism and signalling pathways, tumor cells demonstrate elevated levels of ROS. Tumor cells possess a robust antioxidant ability facilitated by the glutamine metabolic pathway, enabling them to evade detrimental consequences like senescence, apoptosis, or ferroptosis to maintain their continued survival. The most important means by which glutamine metabolism regulates ROS levels is through the production of glutathione, a tripeptide (Glu-Cys-Gly), a powerful antioxidant that eradicates ROS and counteracts peroxyl radicals, the production of which is highly dependent on glutamine [76]. The specific process involves exchange of glutamate produced from glutamine for cystine (which is rapidly reduced to intracellular cysteine) via the SLC7A11 and SCL3A2), which in turn can synthesize glutathione with glutamate, cysteine, and glycine (Fig. 1) [68].

Glutamine can promote glutathione production by regulating mTOR. Moreover, when malic acid derived from glutamine is catalysed by malic enzyme to produce pyruvate [43, 77, 78], and through serine generation in glycine in the presence of SHMT [79, 80]. In essence, both these processes lead to production of NADPH to regulate ROS balance in the cell.

Glutamine has been closely linked to the synthesis of the aforementioned substances in tumor cells and the production of ATP. Overall, glutamine can promote the accumulation of fatty acids, amino acids, and nucleotides at levels necessary for maintaining tumor cell proliferation by directly contributing carbon and nitrogen, indirectly producing reducing equivalents and thus stimulating certain important signalling pathways (Fig. 2).

## Oncogenes and glutamine metabolism

The excessive proliferation of tumor cells often requires a continuous supply of various substances that can effectively promote the metabolic reprogramming of glutamine depletion, which is closely related to dysregulation caused by different oncogenes. Dysregulation caused by oncogenes can alter multiple important sites of glutamine amide catabolism and anabolic metabolism in tumor cells, thus facilitating tumor cells to obtain large amounts of the nutrients from the surrounding extracellular microenvironment to actively support the tumor growth and metastasis in a destructive manner [81, 82]. This section focuses on the role of *c-myc, KRAS*, *HIF* and *p53* genes (Fig. 1).

### C-myc

*c-myc* increases the dependence of tumor cells on exogenous glutamine and enhances glutaminolysis to promote the TCA cycle [83], which in turn can increase the synthesis of nucleotides, proteins and other substances, thus providing a material basis for tumor cell development, metastasis and drug resistance [38, 84, 85]. After extensive research, *c-myc* was shown to increase the expression of transport proteins or enzymes related to glutamine metabolism such as GLS1, SLC1A5, and SLC7A11, thereby increasing glutamine uptake and metabolism rates [39, 85–88]. For example, in hepatoma cells, *SLC1A5* mRNA expression was reduced by 20–30% and glutamine uptake was attenuated by 40% in *c-myc*^+/−^ mice and cells in comparison to the levels in *c-myc*^+/+^ mice [87]. By regulating *SLC1A5* transcription, *c-myc* can modulate mTORC1 kinase activation, triggering a series of mTORC1-related effects [87]. *c-myc* can act on *SLC7A11* to promote glutamine excretion, in exchange for an increase in intracellular cysteine, which in turn was used for glutathione synthesis, thus protecting tumor cells from oxidative damage [85, 88]. In addition, glutathione synthesis (serine hydroxymethyltransferase-2), and nucleoside synthesis (Ribonucleotide Reductase Regulatory Subunit M2 and Adenylosuccinate lyase) have been linked to glutamine metabolism, and glutamate dehydrogenase 1(*GLUD1*) and *GOT2* are among the identified *c-myc* target genes [39, 83, 86].

In addition to regulating key enzymes in glutamine metabolism, *c-myc* can also induce an increase in the levels of various oncogenic metabolites to regulate the progression of tumor development. 2-Hydroxyglutaric acid is an α-KG-derived oncogenic metabolite that causes epigenetic alterations and activation of the mTOR signalling pathway. Its production has been associated with *c-myc* overexpression, which contributes to the progression asymptomatic precursor plasma cell (PC) malignancies into symptomatic multiple myeloma (MM) [84]. Interestingly, *c-myc* plays a crucial role in orchestrating the sequential activation of glycolysis and glutaminolysis, as well as FA and PG synthesis (FA synthesis follows glycolysis and glutaminolysis), thereby allowing tumor cells to balance access to the nutrients with the stoichiometric production of substances [86]. Moreover, the regulation of SLC1A5 by *N-myc downstream regulated gene 2* (*NDRG2*), a gene regulated downstream of N-myc, is also mediated by *c-myc* [89].

*c-myc*, which is overexpressed in a variety of tumor cells, is regarded as one of the most prevalent and aggressive oncogenes and is often associated with chemotherapy resistance and poor clinical prognosis in cancer patients. Hence, the findings discussed here provides valuable insights into the mechanisms behind this resistance and offers potential pathways for future cancer treatments, particularly those involving the targeting of both *c-myc* and glutamine metabolism. It can be concluded that in order to achieve more better therapeutic outcomes, it may be advantageous to target both *c-myc* and glutamine metabolism.

### KRAS

Similar to other tumor cells, tumor cells driven by the *KRAS* gene display high levels of glycolytic activity, thereby enhancing glutamine anabolism. Interestingly, the carbon as well as nitrogen molecules in glutamine have been implicated in the generation of amino acids, nucleotides and glutathione for growth maintenance and ROS inhibition, thus promoting tumor cell survival and proliferation [90–92].

*KRAS* has the ability to increase the expression of the glutaminase amino acid reverse transporter protein SLC7A5, which exports glutamine in exchange for amino acids needed for various cellular functions such as protein synthesis [93, 94]. *KRAS* can promote the accumulation of succinate in the form of α-KG in a SLC25A22 (a mitochondrial glutamate carrier that mediates glutamine catabolism)-dependent manner. This mechanism promoted DNA methylation, caused activation of β-catenin and increased expression of *LGR5* as well as augmented proliferation of tumor cells. Additionally, it induced the acquisition of stem cell-like features and rendered resistance to 5-fluorouracil treatment in colorectal cancer cells [94]. Importantly, *KRAS* also amplified macropinocytosis by tumor cells, promoted the rate of extracellular protein transport into the cells, and increased glutamine production after proteolytic degradation of various internalized proteins [95].

*KRAS* also maintains redox homeostasis through the metabolism of glutamine. On the one hand, *KRAS* upregulates Nrf2 expression, which in turn can induce SLC7A11 expression [40]. This mechanism involves the activation of Nrf2, which sensitizes *KRAS*-driven tumor cells to inhibit glutaminase activity [96]. Moreover, *KRAS* can also directly transactivate the SLC7A11 promoter by cooperating with the Ets-1 transcription factor downstream of the Ras-Raf-Mek-Erk pathway and ATF4 [97]. Upregulation of SLC7A11 can lead to increase in cystine uptake, which in turn can promote glutathione synthesis. Furthermore, in *KRAS*-driven PDAC, aspartate obtained from glutamine was transported to the cytoplasm (aided by mitochondrial uncoupling protein 2) and transformed into oxaloacetate by *GOT1* [54]. This oxaloacetate was further converted into malate and pyruvate, a process that was facilitated and concurrently increased NADPH/NADP^+^ levels to ensure cellular redox balance [90]. Additionally, *KRAS* also influenced the regulation of another transaminase, *GOT2* [43, 98].

A considerable number of tumors exhibit *KRAS* mutations, but at the stage of discovery, it is difficult to directly target *KRAS* proteins to improve treatment, and some researchers even consider *KRAS* to be an undruggable target [99]. However, as discussed above, tumor cells after *KRAS*-driven metabolic reprogramming can be blocked in the S or G2/M phase due to glutamine deprivation and therefore can be effectively targeted by transaminase inhibitors or S-phase-specific toxic compounds during the therapy. Moreover, it is possible to develop therapeutic interventions that address the association between *KRAS* and redox balance. In conclusion, the limitation in targeting *KRAS* can be overcome by manipulating glutamine metabolism, providing a potential avenue to treat challenging oncogenic mutations other than those in *KRAS*.

### HIF

Under hypoxic conditions, glutamine metabolised in tumor cells can enter the TCA cycle and shift glutamine metabolism from an oxidative to a reductive carboxylation pathway [70, 100]. Tumor cells primarily depend on α-KG derived from glutamine to generate acetyl coenzyme A, citrate, and lipids through reductive carboxylation, [70, 71, 101]. This process could be induced by transactivation of the gene encoding pyruvate dehydrogenase kinase 1 (*PDK1*) by HIF-1α, thus reducing the amount of pyruvate entering the TCA cycle [100]. Remarkably, human B-cell lymphoma cells predominantly engage in glutamine metabolism, facilitating the distribution of TCA metabolites, while reductive carboxylation remains minimal in this process [102].

HIF-1α and HIF-2α, the two best-known hypoxia-activated transcription factors, share similar biochemical characteristics but exert differential effects on tumor cells in hypoxic environments. They exhibit similarities such as their ability to control specific enzymes or transporter proteins involved in glutamine metabolism and the expression of *c-myc*. However, their mechanisms of action and outcomes differ indicating the necessity to examine these proteins individually [103, 104].

HIF-1α stimulates the expression of genes involved in glycolysis, leading to an increased conversion of glucose to lactate. This adaptation in tumor cells enhances their reliance on glutamine as a crucial source for biosynthesis, enabling them to effectively adapt to the hypoxic environment [100]. HIF-1α plays a role in promoting the transcription of various glutamine transporter proteins such as GLS1, SLC7A5, SLC38A2, and SNAT2, which are involved in regulating glutamine metabolic processes [105–107]. Similar to other oncogenes, HIF-1α increases HIF-1α stimulates SLC7A11 expression, facilitating the exchange of glutamine with cystine, elevating glutathione levels within tumor cells, preventing the accumulation of ROS within tumor cells and impeding the occurrence of ferroptosis in tumor cells [108, 109]. These effects have been demonstrated in the peritoneal metastasis of gastric cancer, potentially after lncRNA-PMAN was regulated by HIF-1α, to promote the cytoplasmic distribution of ELAVL1, thereby increasing the stability of SLC7A11 mRNA [109]. During glutamine catabolism, HIF-1α binds the promoter of *GLUL*, leading to increased transcription of *GLUL* under hypoxic conditions and after intracellular glutamate accumulation. This facilitates glutamine uptake and catabolism, but these processes can significantly reduce the sensitivity of human lung cancer cells to cisplatin and render tumor cells resistant to cisplatin [110].

However, it has been suggested that HIF-1α may inhibit the proliferation of tumor cells by regulating the glutamine catabolic process. For instance, in clear cell renal cell carcinoma, HIF1α can directly inhibit aspartate biosynthesis by acting on *GOT1* and *GOT2* to suppress both glutamine oxidation and glutamine reduction pathway production of aspartate. This concurrent inhibitory action can result in a significant decrease in the total amount of intracellular aspartate following HIF1α activation [111]. The simultaneous effect of HIF1α on *GOT1* and *GOT2* can also lead to the inhibition of the malate-aspartate shuttle (since *GOT1* can convert aspartate generated in the presence of *GOT2* to oxaloacetate in the cytoplasm) [112]. Due to the diminished capacity of tumor cells to take up aspartate compared to other metabolites [42, 52, 55, 113], this counteracting effect of the glutamine production of aspartate by HIF-1α can significantly inhibit the proliferation of tumor cells. HIF1-α regulates glutamine metabolism by inhibiting the activity of α-ketoglutarate dehydrogenase (α-KGDH), an essential enzyme in mitochondria, while simultaneously increasing the levels of α-KG, which in turn drives the inverse reaction of isocitrate dehydrogenase [114]. In addition, HIF-1α hinders the transcriptional ability of *c-myc*, specifically by competing with *c-myc* to bind sp1, causing a certain degree of cell cycle arrest and decreasing the proliferation rate of tumor cells [103, 115]. However, in most cases, HIF-1 is believed to have a tendency to facilitate the growth of cancer cells and its inhibitory effect can be offset by other compensatory pathways in tumor cells.

Similar to HIF-1α, HIF-2α can induce the expression of SLC38A2, SNAT2, SLC1A5 variant, GLS1, and SLC7A5, which can facilitate the rapid growth of tumor cells [106, 116–118]. Notably, in pancreatic cancer, HIF-2α-induced overexpression of the SLC1A5 variant leads to an augmented production of ATP and glutathione synthesis when induced by glutamine. This process ultimately results in pancreatic cancer cells developing resistance to gemcitabine treatment [116]. Several studies have shown that HIF-2α can enhance the expression of *c-myc* to promote the proliferation of tumor cells in hypoxic environments [104, 119]. During glutamine catabolism, HIF-2α knockdown effectively inhibited glutamine uptake and GOT1 expression through a *c-myc*-dependent pathway [119, 120]. Furthermore, a functional lactic acid/HIF/2α- *c-myc* signalling pathway has been identified in tumor cells, and lactic acid in the in vitro environment not only was able to stabilize HIF-2α but also activated *c-myc* [121]. HIF-2α stabilized *c-myc* expression during this process leading to increased glutamine metabolism by upregulating SLC1A5 and *GLS1* levels through its effect on *c-myc* [121]. The regulation of *c-myc* by HIF-2α could also be potentially realized by regulation of the interactions between *c-myc*/Sp1, *c-myc*/Miz1 and *c-myc*/Max [104]. Notably, Jhudit’s study highlighted the interconnection between lactate metabolism and glutamine catabolism in tumor cells, presenting potential avenues for investigating the relationship between these factors in oxidized tumor cells in future studies [121]. In addition, HIF2α has been associated with mTOR. The expression of SLC7A5 is increased and mTORC1 activity is enhanced by HIF2α through specific binding to the proximal promoter of SLC7A5 [117]. Moreover, HIF-2α enhances glutamine metabolism by activating the PI3K/mTORC2 pathway, leading to an increase in the NADPH/NADP^+^ ratio and counteracting the effects of ROS [120].

Additional studies have indicated that the coordination between HIF-1α and HIF-2α is essential in controlling the expression of the *GRIA2* and *GRIA3* genes, which are responsible for encoding glutamate receptors [122]. The activation of SRC family kinases and subsequent signalling pathways that promote cancer cell proliferation, apoptosis resistance, migration and invasion in different cancer cell lines are triggered by the binding of glutamate to receptors [122].

In conclusion, the effects of HIF-1α and HIF-2α on glutamine metabolism in tumor cells varies, as HIF-2α opposes HIF-1α and supports the progression of tumor cells. Additional studies can provide more insights into the relationship between HIF-1α and HIF-2α and their impact on glutamine metabolism. This knowledge advancement holds the potential for enhancing the development of more effective therapeutic drugs that can specifically target HIFs in cancer treatment.

### p53

When tumor cells undergo glutamine starvation, it triggers the activation of p53, leading to stimulation of multiple mechanisms that allow the tumor cells to adapt and survive under nutrient-deficient conditions. Under both stress and nonstress conditions, p53 binds to the *GLS2* promoter, and an elevated *GLS2* level can result in enhanced mitochondrial respiration and ATP production [123, 124]. Furthermore, the promotion of glutamine metabolism by p53 leads to an elevation in glutathione levels, thereby decreasing reduces ROS levels in tumor cells, thus controlling the apoptosis of tumor cells [123, 124]. This role of *GLS2* in turn promotes the ability of p53 to effectively protect the cells from accumulated genomic damage and allows them to survive after mild and repairable genotoxic stress [123, 124]. In addition, as a direct downstream target of p53, *GLS2* mediates the function of p53 and can inhibit metastasis by suppressing Rac1 signalling pathway [125]. During glutamine deprivation, SLC1A3 (an aspartate/glutamate transporter protein) acts as a key mediator of p53 function of supporting of cell survival and proliferation. It enables the use of aspartate as a source of energy to maintain the activity of ETC and TCA cycle in the absence of extracellular glutamine. Promoting de novo synthesis of glutamate, glutamine and nucleotides to restore lost cell viability [56]. Additionally, in the case of glutamine deprivation, p53 activation causes transcriptional upregulation of solute-like carrier family 7, member 3 (Slc7a), which encodes a protein that is responsible for transporting cationic amino acids across the cell’s membrane, resulting in elevated levels of intracellular arginine [126]. Increased arginine contributes to the sustained activation of mTORC1, which subsequently alters the metabolism of glutamine through the action of mTORC1 [127, 128].

The transcription factors p63 and p73, both belonging to the p53 family, play a significant role in regulating *GLS2*. The transcription factor p63 is essential for several biological processes, including the development and maintenance of epidermal tissue and tumorigenesis [129]. Interestingly, in the primary and tumor cell lines, the TAp63 isoform of p63 can directly bind to the shared p53/p63 DNA binding sequence within the *GLS2* promoter region, thus increasing *GLS2* expression to regulate the cellular metabolism [130]. p73 controls the transcription of *GLS2*, a key enzyme in glutamine catabolism. This facilitates the conversion of glutamine to glutamate, which subsequently promotes the serine biosynthesis process. Therefore, serine and glutamate are readily accessible to produce GSH [131, 132].

### Other genes

Furthermore, apart from these stellar genes, various other factors play a role in regulating glutamine metabolism. The oncogenic form of *PIK3CA* increases the glutamine dependence of tumor cells by upregulating the expression of mitochondrial *GPT2*, which catalyses the transamination reaction that results in conversion of glutamate and pyruvate to α-KG and alanine [133]. The transcription factor c-Jun, a product of the proto-oncogene *JUN*, plays a vital role in regulating GLS levels. Upon activation, the oncogenic Rho GTPase, a signalling molecule downstream of *c-Jun*, directly binds to the GLS promoter region. This interaction enhances GLS gene expression, which not only leads to elevated mitochondrial glutaminase activity but also to increased dependence of tumor cells on glutamine-mediated anaplerosis [134]. CD44 is an extracellular matrix hyaluronan protein that enhances the membrane stability of SLC7A11. The presence of elevated CD44 levels in gastrointestinal tumor cells leads to increased synthesis of GSH and stronger defences against oxidative stress [135]. The reason could be related to CD44v stabilizing SLC7A11 expression at the plasma membrane and the subsequent mediation of Xc-system on cystine uptake to promote GSH synthesis [135, 136]. The Rb protein is a tumor suppressor that is dysregulated in most human cancers. The disruption of global Rb activity led to a significant rise in the absorption and utilization of 13C-glutamine, resulting in incorporation into glutamate and TCA intermediates. This phenomenon can be attributed to upregulated expression of the glutamine transporter *ASCT2* and the activity of *GLS1*. The Rb-controlled transcription factor E2F-3 can alter glutamine uptake by direct modulating *ASCT2* mRNA as well as protein expression, and E2F-3 was observed to associate with the *ASCT2* promoter [137]. In addition, the tumor suppressor LKB1 was reported to inhibit the growth of tumor cells by regulating HIF-1α [138].

Taken together, these findings indicate a close relationship between various oncogenes and key enzymes in glutamine metabolism. Hence, it is evident that through pharmacological modulation of these genes and associated enzymes, innovative therapeutic agents targeting the glutamine metabolic process can be developed.

## Glutamine and mTORC1

mTOR, a widely conserved serine/threonine protein kinase, comprises two complexes, mTOR complex 1 (Raptor-mTOR, mTORC1) and mTOR complex 2 (Rictor-mTOR, mTORC2). Among these two kinases, mTORC1 plays a key role in maintaining metabolic homeostasis, protein and lipid synthesis, glycolysis, mitochondrial biosynthesis, and lysosomal function by sensing various stimuli, such as amino acids, growth factors, and oxygen, thereby regulating activity of transcription factor and directly modulating translation processes. In addition, it can regulate glucose metabolism and nucleotide synthesis, as well as proteasome assembly and autophagy [139]. In essence, mTORC1 affects the various metabolic processes related to cell proliferation, growth and metabolism. By integrating these functions, mTORC1 serves as the primary regulator of metabolic reprogramming in tumor cells [140].

**Fig. 3:**
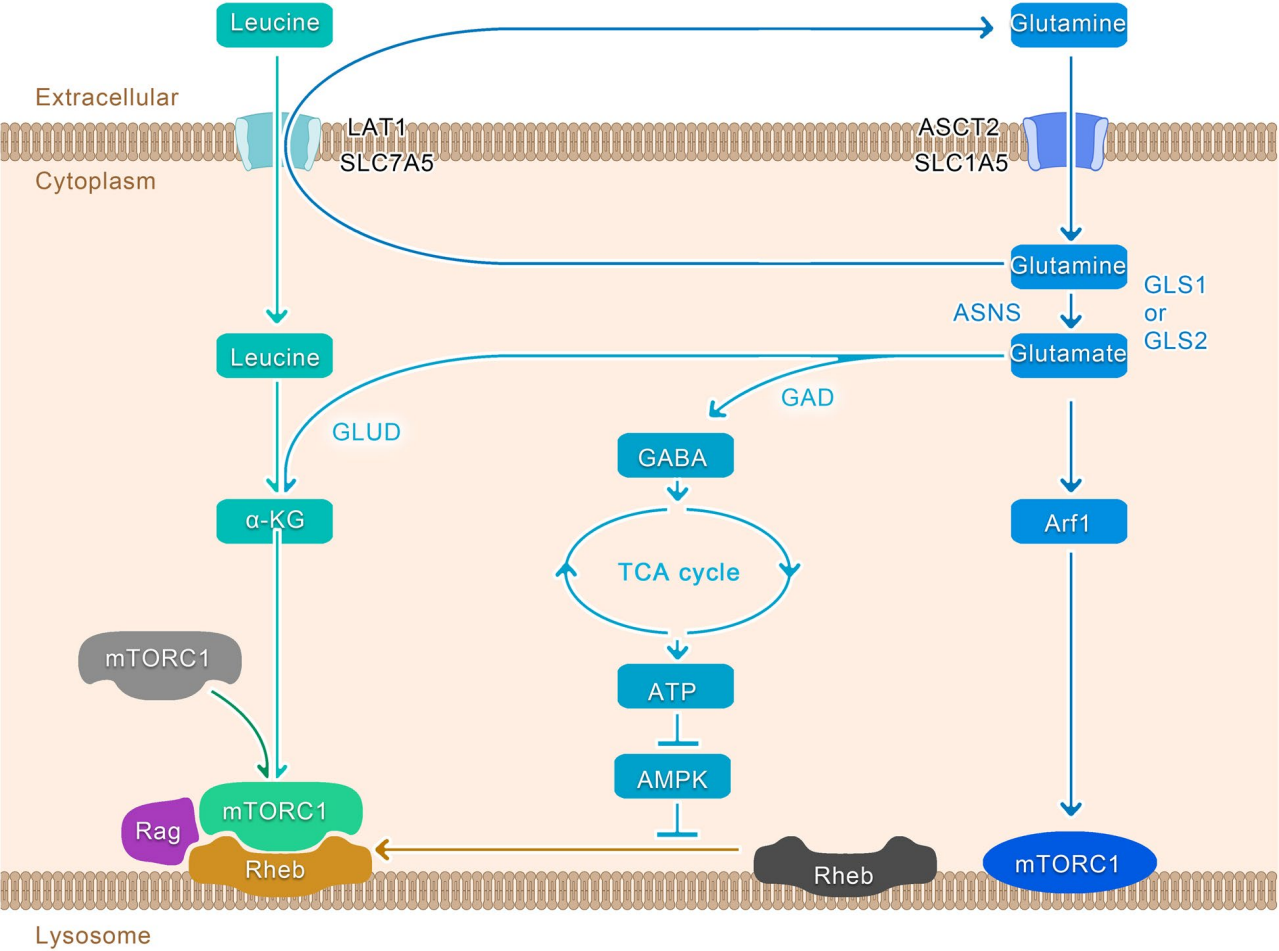
Glutamine and mTORC1. In the Rag-dependent pathway, glutamine is activated by the action of *GLS1* or *GLS2* to produce glutamate, which in turn can generate α-KG and, together with leucine acquired from glutamine exchange through LAT1, induces Rag-mediated recruitment of mTORC1 to the lysosomal surface. When glutamine catabolism is inactivated, tumor cells can synthesize glutamate via ASNS-mediated glutamine conversion, which in turn can generate ATP, thereby inhibiting the AMPK pathway and completely activating mTORC1 on the lysosomal surface, a process mediated by the mTORC1 coactivator Rheb. Moreover, glutamine can also regulate mTORC1 in a non-Rag-dependent manner via Arf1

Glutamine can potentially control the growth of tumor cells by regulating mTORC1 [87, 93], and deprivation of glutamine in tumor cells can significantly decrease the activity of mTORC1 [141]. During the tumor cell proliferation, glutamine catabolism can activate mTORC1, leading to abnormal inhibition of autophagy and subsequent apoptosis [140, 142, 143]. This death pathway has been named as glutamoptosis [144]. Recent studies have discovered numerous glutamine-activated mTORC1 pathways within tumor cells, which are responsible for promoting unlimited cell growth and proliferation [128, 145, 146]. One such pathway is the Rag-dependent pathway. As shown in Fig. 1, glutamine is exchanged with essential amino acids, including leucine, via SLC7A5 in tumor cells [147]. This interaction between glutamine and leucine has a significant impact on various cellular functions, including growth and autophagy by enhancing the glutaminolytic process, particularly α-KG production and Rag-mediated recruitment of mTORC1 to the lysosomal surface, thereby activating mTORC1 [142, 143, 148]. This Rag-dependent regulation is possibly influenced by the lysosomal amino acid transporter protein SLC38A9 and the leucine sensor Sestrin 2 [145, 146, 149–152]. However, the mechanisms through which α-KG can affect Rag during this process remain unclear. Another pathway does not involve the Rag-dependent form as glutamine in tumor cells can regulate mTORC1 via ADP-ribosylation factor 1(Arf1) [148]. Similarly, glutamine-derived asparagine can activate mTORC1 through *Arf1* [128] (Fig. 3). Furthermore, when glutamine catabolism is inactivated, tumor cells can produce glutamate via asparagine synthetase (*ASNS*)-mediated glutamine, which in turn can generate ATP, thereby inhibiting the AMPK pathway and completely activating mTORC1 on the lysosomal surface, a process primarily mediated by the mTORC1 coactivator Rheb [144]. This pathway explains the lack of significant efficacy observed when solely targeting *GLS1* as a potential anticancer strategy [61, 144].

In contrast, an mTORC1/SIRT4/GDH/glutamine axis has been identified in cells, and mTORC1 can effectively promote cell proliferation by inhibiting the transcription of SIRT4, a sirtuin located in the mitochondria. This suppression leads to the activation of GLUD-mediated glutamine anaplerosis. Notably, the expression of SIRT4 is reduced particularly in lung cancer, and is associated with patient prognosis [153, 154]. In addition, mTORC1 plays a key post translational role in stabilizing GS by preventing its ubiquitination and proteasomal degradation, thus regulating glutamine production, which is beneficial under conditions of glutamine deprivation [155, 156] (Fig. 1).

## Glutamine inhibitors and tumor therapy

The key role of glutamine metabolism in cancer cells has been established under both in vitro and in vivo settings, making it an attractive target for anticancer therapy and many researchers are developing strategies to target glutamine metabolism as a potential therapeutic target for cancer [157, 158]. Most recently developed drugs have been found to modulate glutamine metabolism by inhibiting the initial stages of glutamine catabolism, although none have been used for cancer treatment in the clinic [159]. Currently, researchers have developed small molecule inhibitors against glutaminase, including CB-839, 6-Diazo-5-oxo-l-norleucine(DON), Bis-2-(5-phenylacetamido-1,3,4-thiadiazol-2-yl) ethyl sulfide (BPTES), etc. However, most of them are still in the preclinical stage of “tool compounds”, or the drugs have a certain degree of toxicity that makes it difficult to use them in the clinic [160–162].

CB-839 is a selective, reversible, orally active GLS1 inhibitor that shows that demonstrates favourable characteristics in terms of pharmacokinetics, solubility, and stability. CB-839 is currently undergoing preclinical trials and has demonstrated good safety and efficacy. Moreover, CB-839 can treat tumors synergistically in combination with other targeted tumor inhibitors, making it the most likely *GLS1* inhibitor for clinical use in the future. For example, CB-839 is used with various drugs such as paclitaxel, nivolizumab, or capecitabine to treat tumors and has entered Phase II clinical trial [160]. The other *GLS1* inhibitor, BPTES, can only be used as a preclinical tool because of its poorer solubility and biological activity [162]. Since *GLS2* is a tumor suppressor, relatively few therapeutic studies related to *GLS2* have been reported in the literature [163, 164].

The glutamine transporter proteins SLC1A5, SLC7A11, and GLUD can be inhibited by various compounds such as γ-FBP, Sulfasalazine, and epigallocatechin-3-gallate (EGCG), respectively [165–167]. Researchers have developed a variety of drugs targeting SLC1A5, an essential glutamine transporter protein in tumour cells. Examples include L-γ-Glutamyl-p-nitroanilide (GPNA), V-9302, and γ-FBP [12, 165, 168]. Among them, GPNA is widely used as a tool compound to inhibit SLC1A5 [165]. However, GPNA also inhibits the transport of other amino acids [169]. And another SLC1A5 inhibitor, V-9302, still functions when SLC1A5 is knocked down [12]. Therefore, no SLC1A5 inhibitors with high specificity have been identified or designed, and SLC7A11 and GLUD inhibitors have not yet been applied to clinical oncology because of toxicity or efficacy as tool compounds [167, 170].

In terms of drug development, the presence of high cytotoxicity, weak binding selectivity, or poor aqueous solubility in certain drugs like DON and BPTES poses significant obstacles for their inclusion in clinical studies [171, 172]. These problems could be potentially solved by synthesizing prodrugs [158, 162], by designing nanocapsules or even by using novel drug-delivery robots to target drug delivery to the tumor tissues [173]. Some researchers have designed JHU083, a prodrug of the glutamine antagonist DON, which can preferentially activate enzymes enriched in the tumor microenvironment with some selectivity [174]. In hormonal mice, the drug blocked tumor cell metabolism while reducing hypoxia, acidosis and nutrient depletion, thereby promoting natural anti-tumour T-cell responses. The study also showed that the combination of JHU083 with programmed cell death protein 1 (PD-1) was effective in improving anti-tumour effects compared to PD-1 alone [174]. This demonstrates that glutamine metabolism can serve as a metabolic checkpoint for tumor immunotherapy. In a recent study, researchers successfully used tumor-derived exosomes to target AIEgens and proton pumps, resulting in significant inhibition of tumor growth. Additionally, they found that this approach promoted immunogenic tumor cell death, demonstrating the potential of exosomes as powerful tools for targeting glutamine metabolism [175].

Future treatment strategies that target glutamine metabolism should primarily focus on impairing glutamine utilization by tumor cells while maintaining the physiological requirements of the normal tissues. Furthermore, in light of increasing tumor resistance, it may prove more beneficial to use combination therapies or block multiple metabolic pathways simultaneously rather than relying on a single agent [176]. It has been established that different types of tumor cells exhibit varying levels of reliance on glutamine, and improved therapeutic outcomes could potentially be achieved by specifically targeting glutamine metabolism inhibitors towards tumor cells that heavily depend on glutamine [51]. One way to address the variability of glutamine dependence is to identify novel biomarkers that can accurately identify cancer patients who would benefit from targeted therapy focusing on glutamine metabolism. For example, a preclinical tool based on fluorine 18-(2S,4R)-4-fluoroglutamine (FGln) PET has recently been developed for radiotracer imaging in the humans to identify abnormalities associated with glutamine metabolism in patients [177]. It is expected that metabolic imaging technology to find its way into clinical applications due to its ongoing advancements Table 1. However, there is a lack of research on the potential compensatory effects of tumor cells when the metabolism of glutamine is blocked, which can impact the effectiveness of therapy [178].

**Table 1:** Drugs target glutamine metabolism in cancer

Classification	Drug		Current Status	References
GLS1 inhibitors	BPTES		Poor water solubility and low bioavailability in vivo; preclinical tool;	[38, 39, 162, 179]
	968		Preclinical tool;	[180–183]
	CB-839 (BPTES derivatives))		Clinical trials for various periods;	[39, 40, 184–186] NCT02071862, NCT02071888, NCT02071927, NCT02771626
	CB-839 Selenadiazole derivatives	CPD-20, CPD-23	Preclinical tool	[187]
		Physapubescin I		[188]
	UPGL00004		Preclinical tool	[189, 190]
SLC1A5 inhibitors	γ-FBP		Preclinical tool	[165]
	Benzylserine			[191, 192]
	GPNA			[168]
	V-9302			[12]
SLC7A11 inhibitors	Sulfasalazine		FDA approved for arthritis	[166]
	Erastin		Tool compound, induces ferroptosis	[170]
GLUD inhibitors	EGCG		Tool compound	[11, 167], NCT02891538
	Epicatechin gallate(ECG)		Preclinical tool	[193]
	R162		Preclinical tool	[194]
Glutamine mimics	DON		Off-target effects on nucleotide biosynthesis; limited application due to toxicity;	[14, 102, 161]
	Acivicin			
	Azaserine			
	DON Premedication	JHU-083	Preclinical tool; administered in an inert state	[174, 195]
		methyl-POM-DON-isopropyl-ester		[161]
		Nedelcovych-13d		[196]
		DRP-104	In Phase I/II clinical trials	[197] NCT04471415
Glutamine depletion	L-Asparaginase		Limited due to toxicity, FDA approved to treat ALL	[198]

## Conclusions

Glutamine plays a vital role in the survival, growth, as well as energy regulation of tumor cells, and has been one of the most extensively studied nutrients in the field of tumor cell metabolism. Under the influence of metabolic reprogramming, tumor cells are highly dependent on the glutamine metabolic pathway to produce sufficient energy and substances to support their aberrant growth. In this comprehensive review, we describe in detail the uptake, transport, and metabolism of glutamine in tumor cells and the central role played by glutamine in regulating the synthesis of NEAA, nucleotides, fatty acids, as well as ATP. Furthermore, glutamine can act as an antioxidant against ROS by utilizing its derivative, glutathione to maintain the survival of tumor cells. In tumor cells, various oncogenes such as *c-myc*, *KRAS*, *HIF*, and *p53* can augment the glutamine metabolism pathway. At the same time, glutamine possesses the ability to modulate mTORC1, enhancing the reliance of tumor cells on this particular amino acid.

Based on the in-depth understanding of glutamine metabolic pathways, the development of molecular drugs that specifically target glutaminase or transporters offers innovative strategies to combat tumor growth and curb tumor progression. Several new drugs targeting glutamine metabolism are currently being developed. However, heterogeneity in tumor cell metabolism cannot be overlooked. Within an individual cancer patient, tumor cells may exhibit significant differences in their dependence on glutamine. Even within the same tumor tissue, tumor cells can display inevitable metabolic heterogeneity due to clonal evolution. This heterogeneity signifies that the response and effectiveness of glutamine metabolism inhibitors may differ among various cancers. Therefore, many obstacles must be overcome before developing clinically effective drugs. 1. How to identify novel biomarkers that can withstand validation to pair patients with glutamine-dependent tumors with inhibitors of glutamine metabolism. 2. How to effectively inhibit glutamine metabolism in tumor cells while simultaneously blocking the activation of related compensatory metabolic pathways in tumor cells. 3. How to mitigate the off-target effects of glutamine metabolism inhibitors and protect the normal cells against damage while killing tumor cells. 4. How to solve the limitations of currently developed glutamine inhibitors including their cytotoxicity, poor specificity, and poor water solubility. Further exploration of glutamine metabolism and regulatory mechanisms in tumor cells will aid in obtaining a more extensive grasp on the influence of glutamine in tumor growth, consequently providing a better understanding of the relationship between tumor metabolism and tumor progression. Thus, gaining a comprehensive knowledge of how tumor cells reprogram glutamine metabolism could facilitate the development of more effective therapeutics to improve the prognosis of cancer patients.

## Abbreviations

α-KG: α-Ketoglutarate
α-KGDH: α-Ketoglutarate dehydrogenase
Arf1: ADP-ribosylation factor 1
ASNS: Asparagine synthetase
BPTES: Bis-2-(5-phenylacetamido-1,3,4-thiadiazol-2-yl) ethyl sulfide
CAD: Carbamoyl-phosphate synthetase 2, aspartate transcarbamylase, and dihydroorotase
CIC: Citrate carrier
CPSII: Carbamoyl phosphate synthetase II
CTPS: Cytidine triphosphate synthetase
DON: 6-Diazo-5-oxo-l-norleucine
ECG: Epicatechin gallate
EGCG: Epigallocatechin-3-gallate
ER: Endoplasmic reticulum
ETC: Electron transport chain
FGAM: Phosphoribosylformylglycinamidine
GABA: Gamma aminobutyric acid
GAD: Glutamic acid decarboxylase
Gln: Glutamine
GLS: Glutaminase
GLUD: Glutamate dehydrogenase;
GLUD1: Glutamate dehydrogenase 1
GMP: Guanosine monophosphate
GOT2: Glutamic oxaloacetic transaminase 2
GPNA: L-γ-Glutamyl-p-nitroanilide
GPT2: Glutamic pyruvic transaminase 2
GS: Glutamine synthetase
IDH: Isocitrate dehydrogenase
ISR: Integrated stress response
MM: Multiple myeloma
mTORC1: mTOR complex 1
mTORC2: mTOR complex 2
NDRG2: N-Myc downstream regulated gene 2
NEAA: Nonessential amino acid
PC: Plasma cell
PD-1: Programmed cell death protein 1
PDK1: Pyruvate dehydrogenase kinase 1
PRPP: Phosphoribosylpyrophosphate
PSAT1: Phosphoserine aminotransferase 1
ROS: Reactive oxygen species
SHMT: Serine hydroxymethyltransferase
SLC1A5: Solute carrier family 1 neutral amino acid transporter member 5
TCA: Tricarboxylic acid
